# Optimal Timing of Insecticide Fogging to Minimize Dengue Cases: Modeling Dengue Transmission among Various Seasonalities and Transmission Intensities

**DOI:** 10.1371/journal.pntd.0001367

**Published:** 2011-10-25

**Authors:** Mika Oki, Toshihiko Sunahara, Masahiro Hashizume, Taro Yamamoto

**Affiliations:** Department of International Health, Institute of Tropical Medicine, The Global Center of Excellence, Nagasaki University, Nagasaki, Japan; Centers for Disease Control and Prevention, Puerto Rico, United States of America

## Abstract

**Background:**

Dengue infection is endemic in many regions throughout the world. While insecticide fogging targeting the vector mosquito *Aedes aegypti* is a major control measure against dengue epidemics, the impact of this method remains controversial. A previous mathematical simulation study indicated that insecticide fogging minimized cases when conducted soon after peak disease prevalence, although the impact was minimal, possibly because seasonality and population immunity were not considered. Periodic outbreak patterns are also highly influenced by seasonal climatic conditions. Thus, these factors are important considerations when assessing the effect of vector control against dengue. We used mathematical simulations to identify the appropriate timing of insecticide fogging, considering seasonal change of vector populations, and to evaluate its impact on reducing dengue cases with various levels of transmission intensity.

**Methodology/Principal Findings:**

We created the Susceptible-Exposed-Infectious-Recovered (SEIR) model of dengue virus transmission. Mosquito lifespan was assumed to change seasonally and the optimal timing of insecticide fogging to minimize dengue incidence under various lengths of the wet season was investigated. We also assessed whether insecticide fogging was equally effective at higher and lower endemic levels by running simulations over a 500-year period with various transmission intensities to produce an endemic state. In contrast to the previous study, the optimal application of insecticide fogging was between the onset of the wet season and the prevalence peak. Although it has less impact in areas that have higher endemicity and longer wet seasons, insecticide fogging can prevent a considerable number of dengue cases if applied at the optimal time.

**Conclusions/Significance:**

The optimal timing of insecticide fogging and its impact on reducing dengue cases were greatly influenced by seasonality and the level of transmission intensity. We suggest that these factors should be considered when planning a control strategy against dengue vectors.

## Author Summary

Dengue virus infection is a serious infectious disease transmitted by *Aedes* mosquitoes in the tropics and sub-tropics. Disease control often involves the use of insecticide fogging against mosquito vectors. However, the effectiveness of this method for reducing dengue cases, in addition to appropriate application procedures, is still debated. The previous mathematical simulation study reported that insecticide fogging reduces dengue cases most effectively when applied soon after the epidemic peak; however, the model did not take into account seasonality and population immunity, which strongly affect the epidemic pattern of dengue infection. Considering these important factors, we used a mathematical simulation model to explore the most effective time for insecticide fogging and to evaluate its impact on reducing dengue cases. Simulations were conducted with various lengths of the wet season and population immunity levels. We found that insecticide fogging substantially reduces dengue cases if conducted at an appropriate time. In contrast to the previously suggested application time during the peak of disease prevalence, the optimal timing is relatively early: between the beginning of the dengue season and the prevalence peak.

## Introduction

Dengue virus (DENV) infection is a mosquito-borne viral disease of serious health concern in recent decades. More than two-fifths of the global population is considered to be at risk of dengue infection, principally in the tropics and sub-tropics [1]. Increases in dengue epidemics are likely due to the rapid and broad-ranging migration of people and urbanization, which is accompanied by expanded infestation of vector mosquito: *Aedes aegypti* [2].

Clinical manifestations of dengue infection range from a mild febrile form (dengue fever: DF) to severe and sometimes to fatal forms (dengue hemorrhagic fever: DHF and dengue shock syndrome: DSS). Although the case fatality rate of DHF/DSS has been declining [3], such severe forms always require intensive care and fluid management under hospitalization. Consequently, a major outbreak represents a serious burden on medical facilities. Tetravalent dengue vaccines are now under development and have the potential to effectively prevent disease [4]; however, these vaccines are not currently approved for clinical use.

To date, vector control has been the only measure for dengue prevention. In contrast to the substantial progress observed for vaccine development, vector control strategies have shown limited improvement. The major vector control measures conducted in many dengue endemic areas include: 1) fogging ultra-low-volume insecticide particles (insecticide fogging) that target adult mosquitoes; 2) chemical and biological controls for mosquito larvae in the key containers; and 3) larval source reduction. Among those, insecticide fogging has been commonly implemented, but its impact on reducing dengue cases is still controversial [5], [6].

*Aedes aegypti* is a highly domesticated species that tends to rest in locations hidden indoors, making it hard for insecticide to reach adult mosquitoes [6]. Appropriate timing for insecticide application is also under discussion. Fogging in and around the houses of detected dengue cases is recommended by the World Health Organization during the early phase of a disease outbreak, and is practiced in many endemic areas [7]. However, it has been suggested that fogging following case detection is not conducted early enough to prevent virus transmission occurring across a wider area [7], [8].

In recent years, “in-advance” treatment has been proposed. Fogging is sometimes conducted very early in, or even before, the rainy season [9], however the rationale for such in-advance treatment has yet to be established. Newton and Reiter (N&R) [10] reported that based on a mathematical simulation, the strongest effect of insecticide fogging in preventing dengue cases is expected when insecticides are applied several days after the prevalence peak; however, this method had little impact on disease prevention, with only 6.8% of the cases prevented. Many other researchers have referred to this study as evidence of the ineffectiveness of insecticide fogging [11]–[13].

However, the basic assumptions of the N&R model were oversimplified when compared with the real situation in dengue endemic areas. For example, the N&R model did not take into account seasonal fluctuations in climatic conditions, which influence vector population dynamics and viral development within vectors. In addition, the human population was assumed to be completely naïve to DENV, and the magnitude of the outbreak in their simulation was very large, which resulted in 7,651 people out of 10,000 being infected in an outbreak [11]. This phenomenon might be observed in specific situations, like the first dengue outbreak in Easter Island [14], but would not apply to areas where dengue infections are already endemic.

Population immunity is also likely to widely vary in endemic regions. For example, 100% of Nicaraguan children at the age of 16 are seropositive for at least one of the DENVs [15], whereas only 6.5% of junior high school children in Singapore have been exposed to these viruses [16]. Although dengue is endemic in both countries, the transmission intensity appears to be much higher in Nicaragua, resulting in higher immunity levels compared with Singapore.

When assessing the current dengue situation, seasonality and transmission intensity are critical determinants of epidemic patterns that should be taken into consideration when evaluating and optimizing the impact of insecticide fogging. Some studies suggested that the optimal timing and the impact of insecticide fogging might differ from results reported by N&R when also considering seasonality [12], [17]. However, the most appropriate time for insecticide fogging to effectively prevent dengue incidence was not definitively provided in these studies. Thus, we aimed to identify the optimal timing for insecticide fogging and its impact on reducing cases of DENV infection by using a mathematical simulation model of dengue transmission dynamics that included various seasonal settings and transmission intensities.

## Methods

### The model

We used the structure of the N&R model [10] and partly modified it to: 1) add seasonality and 2) produce the endemic state. Equations are presented below. Host population was divided into *S_h_* (susceptible), *E_h_* (exposed), *I_h_* (infectious) and *R_h_* (recovered). Vector population was also divided into *S_v_* (susceptible), *E_v_* (exposed) and *I_v_* (infectious).

Host population
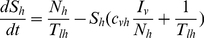
(1)

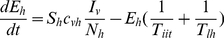
(2)

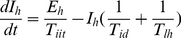
(3)

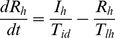
(4)Vector population
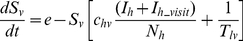
(5)

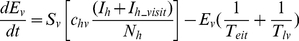
(6)

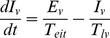
(7)Parameters and parameter values are shown in Table 1.

**Table 1:** Parameter values for the simulations.

Parameter	Symbol	Value	Source
Host population	*N_h_*	10,000	10
Host life span	*T_lh_*	600,060 hours (68.5 years)	10
Intrinsic incubation period	*T_iit_*	5 days	10
Extrinsic incubation period	*T_eit_*	10 days	10
Number of mosquitoes per person	*MPP*	2–15	
Emerging rate of adult mosquitoes	*e*	5,000–37,500/day *	10
Vector life span	*T_lv_*	4 days (wet season)3 days (dry season)	10
Visiting infectious host	*I_h_visit_*	0 in Simulation 1 and 20.001 in Simulation 3 and4	20
Host infection duration	*T_id_*	3 days	10
Effective contact rate, vector to host	*c_vh_*	0.75/day	10
Effective contact rate, host to vector	*c_hv_*	0.375/day	10

*5,000 for Simulation 1–3 (MPP = 2); 7,500, 12,500, 20,000 and 37,500 for MPP = 3, 5, 8 and15 in Simulation 4.

#### Seasonality

We considered both wet and dry seasons. In the real-life situation, seasonal changes in temperature have a great impact on mosquito survival and viral growth, and rainfall influences the availability of larval habitat of the vector [18]–[20]. However, to minimize model complexity, we assumed two seasons that affect only the mosquito lifespan (*T_lv_*). The emergence rate of adult mosquitoes (*e*) was set based on N&R's assumption [10] that carrying capacity was divided by vector lifespan (4 days). When we added seasonality into the model, we assumed that mosquito emergence is not affected by the season and is a constant, which may be true in areas where the dengue vector breed in domestic water containers that do not receive rainwater. The density of adult mosquitoes was calculated as *eT_lv_* at the equilibrium and was higher during the wet season.

#### Insecticide

Single pulse fogging was conducted in all simulations. Fogging was implemented at noon on each day of application. For each insecticide application, 60% of the total mosquitoes were assumed to be killed. No residual insecticide efficacy was included in our model so that, after application, there was no effect on surviving or newly-emerged mosquitoes [10], [17]. N&R assumed a density-dependent recovery rate after insecticide fogging; however, we assumed that the fogging did not affect larval and pupal population. Density dependence was therefore not included in adult population dynamics, and the emergence rate of mosquitoes after fogging was also the same rate at *e*.

### Simulations

Fogging was applied each day from the 1^st^ to the 365^th^ day of the year, during which time, the wet season was assumed to occur at the beginning of the year. The annual number of infected cases was calculated at each application and the day when fogging resulted in a maximum reduction of dengue cases was defined as the optimal day for fogging. The simulation was conducted numerically with a time-step of one hour using Microsoft Excel.

#### Simulation 1: Identical settings to N&R's base case

This simulation was carried out for 1 year using the same settings as N&R's base case simulation [10]; the host population was completely naïve to DENV and the initial value of *I_v_* was set to 1 on the first day of the year. The optimal day for fogging, and the proportion of the prevented cases, were compared with the results from N&R's simulation.

#### Simulation 2: Simulation 1 + seasonality

We added seasonality to the model by changing vector life span (*T_lv_*) for 4 days in the wet season and 3 days in the dry season during the course of the simulation. Wet season duration was set to 4, 5 and 6 months (one month = 30 days). All parameters except *T_lv_* were the same as for Simulation 1. The optimal day for fogging and maximum case reduction were investigated for each wet season in a year.

#### Simulation 3: Simulation 2 + endemic state

This simulation was run for 499 years without any intervention to produce an endemic state. When we simulated dengue transmission over many years with a single initial introduction of DENV, we often found that the prevalence decreased to a very low level during the dry season and never recovered to the visible level during the successive wet season. This is not the case in actual dengue endemic areas, where infected hosts or vectors occasionally enter the system and maintain transmission. To simulate stable seasonal dynamics, the number of infected hosts that temporarily visit an area but are not included in the resident population (*I_h_visit_*) was added into equations 5 and 6. For strict mathematical consistency, *I_h_visit_* should be added to the denominator; however, as we set *I_h_visit_* to be a very small value (0.001) compared with the total population (10,000), it was omitted from the denominator. Therefore, we assumed a constant rate of virus introduction (*I_h_visit_*), which was 0.00001% of the total host population from the first day of the simulation. As the coefficient of variation for annual cases was less than 1% over 10 years (the 490^th^–499^th^ year) in all settings, we considered that the endemic state had been reached in this stage. Fogging was applied in the 500^th^ year, and the optimum day for fogging to minimize dengue cases in this year was calculated.

#### Simulation 4: Simulation 3 + various transmission intensities

These simulations were conducted using higher transmission intensities than in Simulation 2. The number of mosquitoes per person (MPP) was increased from 2 to 3, 5, 8 and 15 for low, moderate, high and very high endemic situations, respectively. The day and proportion of most prevented cases were investigated at each MPP.

## Results

### Simulation 1

**Figure 1:**
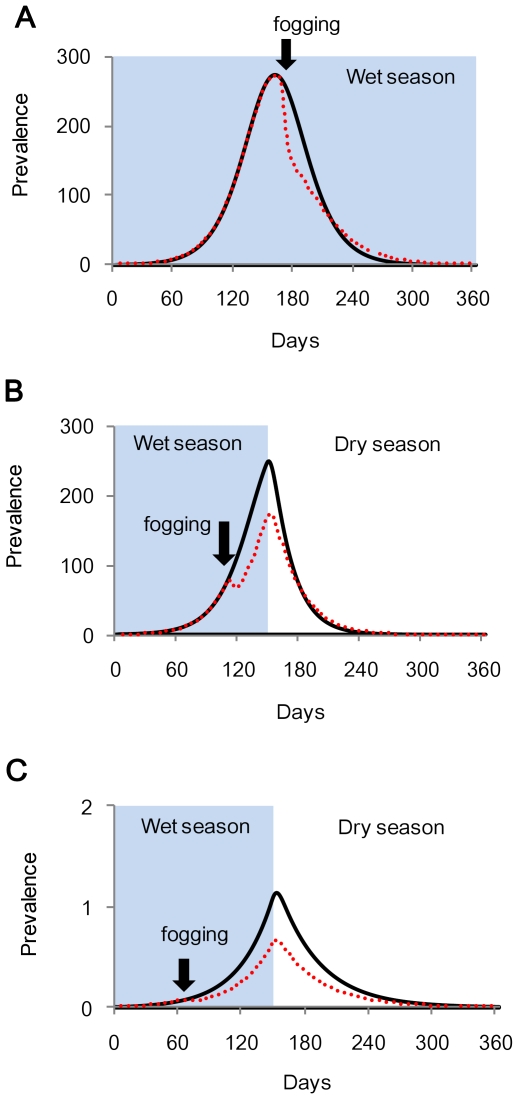
Dengue prevalence with and without optimal insecticide fogging. A: naïve population under non-seasonal condition (Simulation 1), B: naïve population adding 5-month wet season (Simulation 2), and C: endemic state with 5-month wet season (Simulation 3). Black lines indicate untreated epidemics and dotted red lines show epidemics after insecticide treatment. All simulations were conducted using the number of mosquito per person (MPP) = 2. Note that prevalence in Simulation 3 differed from that in Simulation 1 and 2.

Our results were very similar to those obtained for N&R's simulation (Table 2). The maximum reduction in cases was observed when fogging was conducted 6 days after the prevalence peak; however this reduction only amounted to 6.7% of the total cases (Fig. 1A).

**Table 2:** Summary of the simulation results.

Setting	MPP	Wet season (months)	Herd immunity	Day of prevalence peak	No. of annual cases		Prevented cases	Best day of fogging	Difference from the peak
					Without fogging	With fogging			
N&R's base case	2	12	0%	163	7,561.9	7,044.2	6.8%	169	+6
Simulation1	2	12	0%	163	7,616.0	7,120,0	6.7%	169	+6
Simulation2	2	4	0%	125	2,614.8	1,874.0	28.3%	60	−65
	2	5	0%	152	4,788.8	3,793.0	20.8%	112	−40
	2	6	0%	163	6,259.7	5,592.0	14.4%	139	−24
Simulation3	2	4	14.7%	125	21.4	12.8	40.5%	53	−72
	2	5	19.8%	154	29.0	17.3	40.5%	66	−88
	2	6	24.6%	183	35.8	21.4	40.2%	78	−105
Simulation4	3	4	41.7%	125	61.0	35.7	41.4%	44	−81
	3	5	45.5%	154	66.5	39.2	41.1%	58	−96
	3	6	49.0%	184	71.5	42.5	40.6%	71	−113
	5	4	64.8%	125	94.8	56.0	40.9%	40	−85
	5	5	67.1%	154	98.1	58.4	40.5%	56	−98
	5	6	69.2%	184	101.1	60.6	40.0%	68	−116
	8	4	77.9%	125	113.9	68.8	39.6%	41	−84
	8	5	79.4%	154	116.0	70.4	39.3%	57	−97
	8	6	80.8%	184	117.9	72.1	38.8%	70	−114
	15	4	88.1%	125	128.9	81.9	36.4%	45	−80
	15	5	89.0%	154	130.0	82.7	36.3%	63	−91
	15	6	89.7%	184	131.0	84.0	35.9%	79	−105

### Simulation 2

The epidemic magnitude was smaller when the wet season was shorter (Table 2). Dengue incidence generally increased exponentially during the wet season (Fig. 1B), and started to decline rapidly within the few days after the onset of the dry season, during which time, climatic conditions for mosquitoes are unfavorable. Our results showed that the optimal day for fogging was earlier than in Simulation 1 for all wet season durations assessed. The proportion of prevented cases was greater during a shorter wet season (Table 2).

### Simulation 3

In the endemic state, the yearly number of cases was much smaller than that observed in Simulation 1 and 2 (Table 2, Fig. 1C). Optimal timing of fogging shifted to a much earlier time than in Simulation 2, and more than 40% of the cases were prevented during the wet season of any length.

### Simulation 4

Population immunity level also increased with an increase in MPP and the length of wet season (Table 1). In the lower endemic situations (MPP = 3 and 5), a maximum reduction in cases was observed between 81 and 116 days before the prevalence peak, and over 40% of cases were mostly prevented during the wet season of any length. In the higher endemic situations (MPP = 8 and 15), a maximum reduction in cases was also observed earlier than the prevalence peak. The proportion of prevented cases was 35.9–39.6%, which was slightly lower than a MPP of 2, 3 and 5.

**Figure 2:**
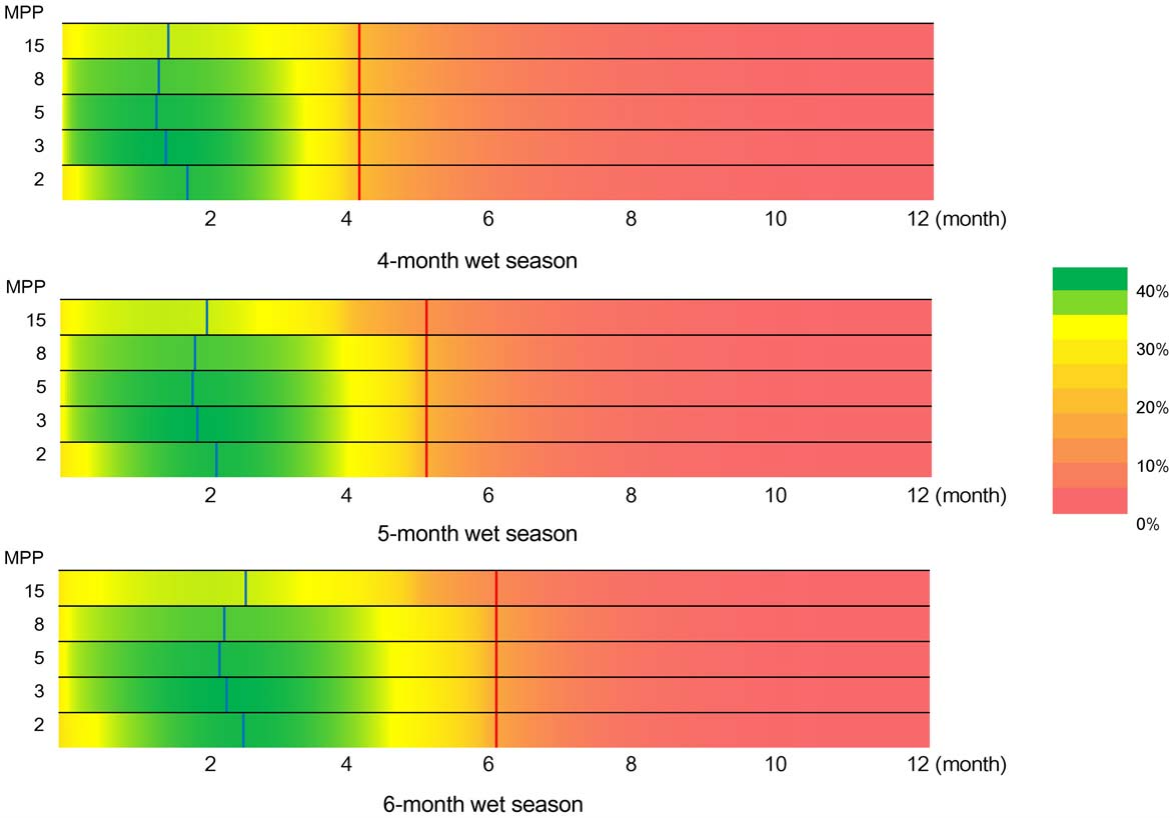
Proportion of cases prevented by single insecticide fogging. MPP is the number of mosquitoes per person. The wet season occurs at the beginning of the year. Blue lines represent the optimal day of fogging and red lines indicate the prevalence peak.

Overall, the most effective time for insecticide fogging was early in the wet season, when over 35% of the cases were prevented at any transmission intensity level. The greatest impact of fogging was observed during shorter wet seasons and for lower transmission intensities. The proportion of cases prevented by fogging on each day of the year is shown in Fig. 2, and the green area indicates the greatest proportion of cases prevented (>40%). The proportion of prevented cases at and after the prevalence peak was not optimal in any settings for an endemic situation.

## Discussion

We successfully developed a model for predicting the most optimal time for insecticide fogging against dengue mosquitoes, which will potentially help reduce the number of dengue cases in endemic regions of the world. By including additional parameters, such as seasonality and disease transmission rates, our model more accurately depicted epidemic outbreaks when compared with the previously published model.

Our simulation results for a naïve population with no seasonal setting were similar to those obtained with the N&R model [10]. The greatest reduction of dengue cases was observed when fogging was conducted several days after the prevalence peak, but the impact was minimal. When climatic conditions are favorable for mosquitoes throughout the year, insecticide fogging only slows down the epidemic curve temporarily even if implemented intensively. After fogging, mosquito populations recover rapidly and transmit DENV to susceptible people. Dengue incidence subsequently continues to increase until population immunity reaches a level at which the recovery rate exceeds the new infection rate. In such a situation, fogging reduced dengue cases when conducted after the prevalence peak by accelerating the natural decline of the epidemic.

When we considered seasonality, the results were completely different. The optimum timing for insecticide fogging shifted earlier than the prevalence peak; because it interferes with the exponential epidemic growth at a certain point and prevents the prevalence peak from reaching the original level by the end of the wet season (Fig. 1B). Furthermore, when considering both endemicity and seasonality, the optimum timing for insecticide fogging shifted to an earlier time and the proportion of prevented cases was greater. The period of greatest prevention was observed relatively early in the wet season (Fig. 2, in green).

DENV has four different serotypes that simultaneously circulate in most dengue endemic countries. Such co-circulation of multiple serotypes greatly influences long-term epidemic patterns. We additionally evaluated the optimal timing of insecticide fogging by including the co-circulation of four serotypes in endemic situation. The results indicated that the optimal application was also between the onset of the wet season and the prevalence peak (results are shown in Text S1). Therefore, we suggest that hyperendemicity did not affect our findings.

Our model however, does have some limitations when applying simulations to actual dengue endemic areas, due to the simplification of parameters to understand the overall effects of insecticide fogging. First, our assumption of seasonal change was represented by two different values of mosquito lifespan, which was too simple to describe real seasonal dynamics. However, as we aimed to provide a practical strategy for determining optimal insecticide fogging in general, we prioritized the model simplicity and clearly distinguished the on and off-dengue seasons. Various biological features may fluctuate seasonally and affect dengue epidemics. However, when a year can be divided into the on and off-dengue seasons, temporary reduction of adult mosquito population by fogging in the middle of the on-dengue season would delay epidemic growth and prevent cases (Fig. 1B). Thus, we considered that our simple setting for seasonality can typically represent more complex dynamics in the real world.

Second, since our model was derived from the N&R model [10], and because we aimed to directly compare our simulations with their conclusion, we set the mosquito lifespan assumption to be identical to this previous study. This was originally obtained from a field study carried out in Thailand (four days in the wet season) [21]. In general, the lifespan of *Ae. aegypti* in the field is estimated to be slightly longer than our assumption: 5.3–9.1 days [22]. However, the low vector survival rate in our model did not affect our conclusion because when we simulated with a 10 day lifespan in the wet season and 7.5 day lifespan in the dry season, the optimal timing of fogging was also between the beginning of the wet season and the prevalence peak (results not shown).

Third, our model did not consider spatial heterogeneity. In our settings, the “in advance” treatment did not appear to be the most effective strategy if implemented too early (Fig. 2). However, incase vector populations survived the dry season in limited areas and expanded the distribution range gradually in the wet season, in-advance focal fogging targeting those areas might be the optimal strategy to reduce the first generation of mosquitoes in the season.

Our study also analyzed the effect of insecticide fogging on preventing total cases in a single year, but not the effect on longer-term total cases. When insecticide fogging prevented many cases, it also reduced immunity in the host population. Consequently, the susceptible population would potentially cause even larger epidemics in subsequent years. We should therefore carefully foresee and take action between epidemics after applying insecticide fogging. Furthermore, when insecticide treatment was routinely conducted every year, we should have also considered the development of insecticide-resistance in the vector population [23], which was not included in our model. As insecticide resistance has already become a serious problem in many dengue endemic countries [24], [25], it is important to carefully consider which insecticides can effectively reduce mosquitoes in the target areas on the basis of biological evidence. The spraying method used to allow the insecticides to reach mosquitoes also requires further investigation. Although our results may not show the best strategy for the long-term prevention of dengue epidemics, they should be interpreted as the optimal strategy for the non-regular emergency treatment during major epidemics.

Despite these limitations, our model has a clear practical significance for dengue control in regions where this disease has been endemic for a long time and its epidemic pattern is affected by seasonal climate factors. The optimal timing of insecticide fogging to reduce dengue incidence most effectively is between the onset of the wet season and the prevalence peak, rather than waiting until the peak of a major outbreak occurs.

## Supporting Information

Text S1 Supplementary method and results of the simulations with four DENV serotypes.
(DOC)
