# Supplementary material for: Optimal Timing of Insecticide Fogging to Minimize Dengue Cases: Modeling Dengue Transmission among Various Seasonalities and Transmission Intensities
Source: PLoS Negl Trop Dis. 2011 Oct 25;5(10):e1367. doi: 10.1371/journal.pntd.0001367 (PMC3201920; doi:10.1371/journal.pntd.0001367)
Supplement: Text S1 — Supplementary method and results of the simulations with four DENV serotypes. (DOC) [file pntd.0001367.s001.doc]

Text S1

We also simulated the optimal timing of insecticide fogging when taking into consideration the co-circulation of four DENV serotypes. Results are shown in Table S1.

In this case, host population was classified as follows: susceptible hosts (*S_h1_*: people who have never been infected with DENV, *S_h2_*: people who have had a primary infection and are at risk of contracting a secondary infection, *S_h3_*: people who have been infected twice and have a chance of contracting a third infection, and *S_h4_*: people who have been infected three times and have a chance of contracting a fourth infection, exposed hosts (*E_h1_, E_h2_, E_h3_, E_h4_*: people who are infected for the 1^st^ to 4^th^ time, respectively, but are not yet infectious to mosquitoes), infectious hosts (*I_h1_, I_h2_, I_h3_, I_h4_*: people who are infected for the 1^st^ to 4^th^ time, respectively, and are infectious to mosquitoes), recovered hosts (*R_h1_, R_h2_, R_h3_:* people who have recovered from prior infections), and resistant hosts (*R_h4_*: people who have been infected four times. We assumed that people acquired permanent immunity to a serotype after recovering from a previous infection, and also developed temporal cross protective immunity (*T_cross_*) to other serotypes for 60 days [1]. For a simple approximation of the complex dynamics, we assumed in our model that the four serotypes always have equivalent infectiousness and prevalence. Furthermore, we assumed that mosquitoes that are infected by two or more serotypes are rare and negligible. Under these conditions, hosts that are susceptible to *n* serotypes can be infected by *n*/4 of the total infectious mosquitoes. Thus, we defined *p_1_*-*p_4_* as the proportion of infectious mosquitoes that can potentially infect the susceptible hosts, *S_h1_*- *S_h4_*, which were set to 1.0, 0.75, 0.50, and 0.25, respectively [2]. Changes in each class of human population were modeled using the following differential equations:

(1)

(2)

$\frac{dI_{h1}}{dt}=\frac{E_{h1}}{IIP}-I_{h1}(\frac{1}{T_{id}}+\frac{1}{T_{lh}})$ (3)

(4)

(5)

(6)

$\frac{dI_{h2}}{dt}=\frac{E_{h2}}{IIP}-I_{h2}(\frac{1}{T_{id}}+\frac{1}{T_{lh}})$ (7)

(8)

(9)

(10)

$\frac{dI_{h3}}{dt}=\frac{E_{h3}}{IIP}-I_{h3}(\frac{1}{T_{id}}+\frac{1}{T_{lh}})$ (11)

(12)

(13)

(14)

$\frac{dI_{h4}}{dt}=\frac{E_{h4}}{IIP}-I_{h4}(\frac{1}{T_{id}}+\frac{1}{T_{lh}})$ (15)

(16)

Vector population was also divided into *S_v_* (susceptible), *E_v_* (exposed), and *I_v_* (infectious). Changes in each class of vector population were modeled using the following differential equations:

(17)

$\frac{dE_{v}}{dt}=S_{v}a_{hv}b_{v}\frac{I_{h1}+I_{h2}+I_{h3}+I_{h4}+I_{h\_visit}}{N_{h}}-E_{v}(\frac{1}{EIP}+\frac{1}{T_{lv}})$ (18)

$\frac{dI_{v}}{dt}=\frac{E_{v}}{EIP}-\frac{I_{v}}{T_{lv}}$ (19)

Simulations were conducted with identical settings to those in Simulation 3 and 4 provided in the main manuscript.

Table S1 Optimal timing of insecticide fogging with considering hyperendemicity

| Setting | MPP | Wet season (months) | Herd immunity* | Day of prevalence peak | No. of annual cases | | Prevented cases | Best day of fogging | Difference from the peak |
| --- | --- | --- | --- | --- | --- | --- | --- | --- | --- |
|  |  |  |  |  | Without fogging | With fogging |  |  |  |
| Simulation3 | 2 | 4 | 36.8％ | 125 | 72.5 | 42.1 | 30.3 | 41 | -84 |
|  | 2 | 5 | 47.3% | 154 | 103.8 | 60.6 | 43.2 | 52 | -102 |
|  | 2 | 6 | 54.2% | 184 | 133.1 | 78.2 | 54.9 | 61 | -123 |
| Simulation4 | 3 | 4 | 73.5% | 125 | 239.3 | 139.9 | 99.4 | 29 | -96 |
|  | 3 | 5 | 76.4% | 154 | 261.0 | 153.7 | 107.3 | 42 | -112 |
|  | 3 | 6 | 78.9% | 184 | 281.1 | 166.8 | 114.2 | 52 | -132 |
|  | 5 | 4 | 87.8% | 125 | 375.8 | 226.3 | 149.6 | 27 | -98 |
|  | 5 | 5 | 88.9% | 154 | 388.8 | 235.6 | 153.3 | 40 | -114 |
|  | 5 | 6 | 89.9% | 184 | 401.0 | 245.0 | 155.9 | 51 | -133 |
|  | 8 | 4 | 93.2% | 125 | 452.9 | 284.7 | 168.1 | 29 | -96 |
|  | 8 | 5 | 93.8% | 154 | 461.0 | 290.4 | 170.6 | 76 | -78 |
|  | 8 | 6 | 94.3% | 183 | 468.6 | 298.8 | 169.8 | 57 | -126 |
|  | 15 | 4 | 96.6% | 125 | 512.8 | 353.1 | 159.7 | 42 | -83 |
|  | 15 | 5 | 96.9% | 154 | 517.2 | 356.7 | 160.4 | 62 | -92 |
|  | 15 | 6 | 97.2% | 183 | 521.2 | 362.7 | 158.5 | 83 | -100 |

* People who have been infected by at least one of four serotypes were counted to possess immunity to DENV.

Reference

1. Sabin AB (1952) Research on dengue during World War II. Am J Trop Med Hyg 1: 30-50.

2. Luz PM, Vanni T, Medlock J, Paltiel AD, Galvani AP (2011) Dengue vector control strategies in an urban setting: an economic modelling assessment. The Lancet. 377: 1673-1680.
